# *ABCC6* Gene Analysis in 20 Japanese Patients with Angioid Streaks Revealing Four Frequent and Two Novel Variants and Pseudodominant Inheritance

**DOI:** 10.1155/2017/1079687

**Published:** 2017-08-20

**Authors:** Satoshi Katagiri, Yuya Negishi, Kei Mizobuchi, Mitsuyoshi Urashima, Tadashi Nakano, Takaaki Hayashi

**Affiliations:** ^1^Department of Ophthalmology, The Jikei University School of Medicine, Tokyo, Japan; ^2^Division of Molecular Epidemiology, The Jikei University School of Medicine, Tokyo, Japan; ^3^Department of Ophthalmology, Katsushika Medical Center, The Jikei University School of Medicine, Tokyo, Japan

## Abstract

**Purpose:**

To report the spectrum of *ABCC6* variants in Japanese patients with angioid streaks (AS).

**Patients and Methods:**

This was a single-center cohort study. The medical records of 20 patients with AS from 18 unrelated Japanese families were retrospectively reviewed. Screening of the *ABCC6* gene (exons 1 to 31) was performed using PCR-based Sanger sequencing.

**Results:**

Eight *ABCC6* variants were identified as candidate disease-causing variants. These eight variants included five known variants (p.Q378X, p.R419Q, p.V848CfsX83, p.R1114C, and p.R1357W), one previously reported variant (p.N428S) of unknown significance, and two novel variants (c.1939C>T [p.H647Y] and c.3374C>T [p.S1125F]); the three latter variants were determined to be variants of significance. The following four variants were frequently identified: p.V848CfsX83 (14/40 alleles, 35.0%), p.Q378X (7/40 alleles, 17.5%), p.R1357W (6/40 alleles, 15.0%), and p.R419Q (4/40 alleles, 10.0%). The *ABCC6* variants were identified in compound heterozygous or homozygous states in 13 of 18 probands. Two families showed a pseudodominant inheritance pattern. Pseudoxanthoma elasticum was seen in 15 of 17 patients (88.2%) who underwent dermatological examination.

**Conclusions:**

We identified disease-causing *ABCC6* variants that were in homozygous or compound heterozygous states in 13 of 18 families (72.2%). Our results indicated that *ABCC6* variants play a significant role in patients with AS in the Japanese population.

## 1. Introduction

Angioid streaks (AS; OMIM #607140) are a hereditary retinal disease involving irregular streaks that radiate from the optic disc due to cracking of Bruch's membrane. There are numerous systemic associations with AS, including pseudoxanthoma elasticum (PXE; OMIM #264800), Paget's disease of bone, sickle cell anemia, and Ehlers-Danlos syndrome [1]. PXE is an autosomal recessive disease characterized by the accumulation of mineralized and fragmented elastic fibers in the skin. The disease is the most common disease associated with AS and was reported to be found in 59% of patients with AS [2].

The ATP-binding cassette subfamily C member 6 (*ABCC6*) gene was first reported in 2000 as the cause of PXE [3–5]. Screening of the *ABCC6* gene has been performed in cases with PXE in several ethnic groups [6–10]. In the Japanese population, there are two case reports of *ABCC6* gene analysis in patients with PXE/A [11, 12]. Subsequently, two large-scale studies for *ABCC6* gene analysis have been reported in 54 patients with AS [13] and in 76 patients with PXE [14] and have revealed differences in the *ABCC6* mutation/variant spectrum between the two studies in Japanese patients [13, 14]. For example, although the p.Q378X variant was frequently seen in PXE patients [14], it was never detected in AS patients [13]. In addition, two variants (p.V848CfsX83 and p.Q378X) are frequently found in PXE patients [14], leading to the hypothesis that frequently found variants may also exist in Japanese patients with AS.

The purpose of this study was to determine whether frequent *ABCC6* variants or hotspots exist in Japanese patients with AS. We screened the *ABCC6* gene in 20 patients with AS from 18 unrelated Japanese families, and we identified four frequent variants, two novel variants, and a pseudodominant inheritance pattern.

## 2. Materials and Methods

### 2.1. Medical Records Used in the Current Study

**Figure 1:**
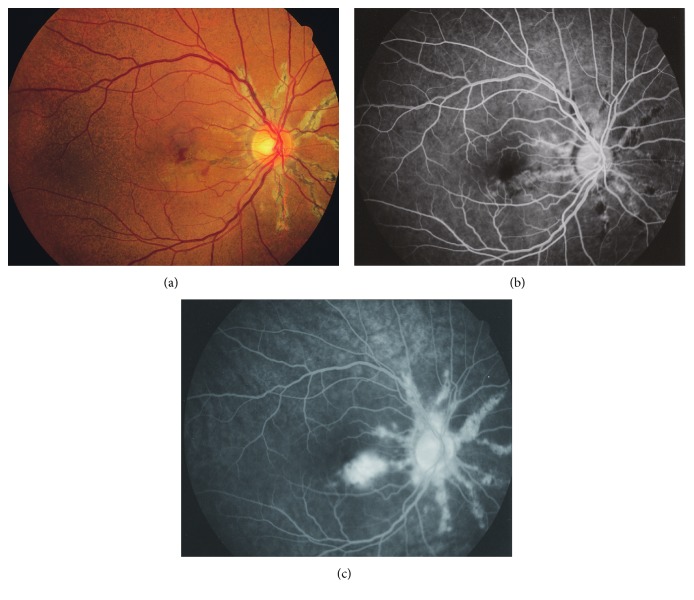
Fundus and fluorescein angiography images of the right eye. Images from a 45-year-old female proband (case 6, JU#0451). The color image (a) shows irregular streaks radiating from the optic disc and a small retinal hemorrhage in the macula. A peau d'orange appearance is also seen temporal to the macula. Fluorescein leakage is observed from the early (b) to the late (c) phases due to choroidal neovascularization.

This was a single-center cohort study. The medical records of 20 patients (11 males and 9 females, aged 16 to 81 years, mean: 53 years) with AS from 18 unrelated Japanese families and eight unaffected family members of these patients were retrospectively reviewed at the Jikei University Hospital from June 2006 to February 2010. There was no consanguinity of parents of all probands (Table 1). The protocol used for this study was approved by the Institutional Review Board of the Jikei University School of Medicine. The protocol adhered to the tenets of the Declaration of Helsinki, and informed consent was obtained from each participant. Ophthalmic and dermatological examinations were performed for the diagnosis of AS with or without choroidal neovascularization (CNV) and PXE; the results are summarized in Table 1. Among the 20 patients with AS, 15 were diagnosed with PXE, two were diagnosed not having PXE, and three were not examined for PXE; eight AS patients were complicated with CNV. Only two patients (cases 3 and 6) with CNV were 50 years old or younger. Fundus and fluorescein angiography images of a representative proband (case number 6, JU#0451) are shown in Figure 1.

### 2.2. Molecular Genetic Studies

Blood samples were obtained from all affected cases and some of their family members. Genomic DNA was isolated from peripheral white blood cells using a Gentra Puregene Blood Kit (Qiagen, Hilden, Germany); the DNA was used as a template for amplifying human *ABCC6* genomic sequences. To analyze the *ABCC6* gene, all 31 exons including the exon/intron boundaries were amplified by PCR with the primer pairs shown in Supplemental Table 1 available online at https://doi.org/10.1155/2017/1079687. The genomic nucleotide sequences of exons 1 to 9 of the *ABCC6* gene were very similar to those of *ABCC6* pseudogenes. In particular, we confirmed that the pseudogenes contained the nonsense variant c.1132C>T (p.Q378X) in exon 9 even in the Japanese population, as was previously reported [6]. For exon 9, to differentiate the real *ABCC6* gene from the pseudogenes, we performed long-range PCR to amplify the 4165 bp region from intron 8 to exon 10 using the following primer pair: forward primer ABCC6-LR3F in intron 8 and reverse primer ABCC6-LR2R in exon 10, which is considered to be a unique exon of the *ABCC6* gene. All PCR reagents, except for the primers, were supplied by Takara-Bio (Shiga, Japan). The PCR amplifications were performed in a DNA Thermal Cycler (PTC-200, MJ Research, Waltham, MA, USA). The PCR products were purified with a QIAquick PCR Purification Kit (Qiagen) and used as templates for sequencing. Both strands were analyzed on an automated sequencer (ABI Prism 3700 DNA Analyzer, Applied Biosystems, Tokyo, Japan). We used BAC clone CIT987SK-A-962B4 (accession number U91318) for genomic DNA and *ABCC6* mRNA (accession number NM_001171.5) sequences from the National Center for Biotechnology Information.

### 2.3. Evaluation of the Identified *ABCC6* Variants

To predict the functional impact of the *ABCC6* variants, we performed three different in silico analyses using the Polyphen-2 (http://genetics.bwh.harvard.edu/pph2/), SIFT (http://sift.jcvi.org), and PROVEAN (http://provean.jcvi.org) programs. To investigate the frequency of the identified variants, we used the Human Genetic Variation database (http://www.hgvd.genome.med.kyoto-u.ac.jp/index.html) and the Exome Aggregation Consortium database (http://exac.broadinstitute.org). We also referred to the Leiden Open Variation Database 3.0 (https://databases.lovd.nl/shared/genes/ABCC6) and the Human Gene Mutation Database (http://www.hgmd.cf.ac.uk) for determining the pathogenicity of the identified variants.

## 3. Results

### 3.1. Pathogenicity of the Identified *ABCC6* Variants

**Figure 2:**
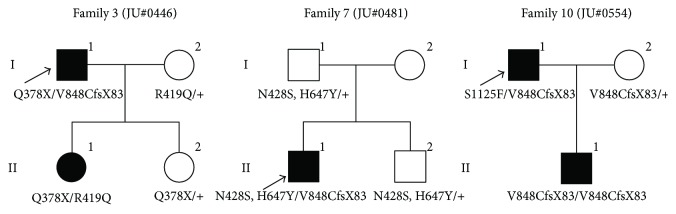
Pedigrees of three Japanese families with angioid streaks (AS). Two novel *ABCC6* variants (p.H647Y and p.S1125F) are found in families 7 and 10. Families 3 and 10 exhibit a pseudodominant inheritance pattern. The identified *ABCC6* variants cosegregated with the patients with AS.

In total, eight *ABCC6* variants were identified as candidate disease-causing variants. The data of these eight *ABCC6* variants are summarized in Tables 1 and 2. Five of these eight *ABCC6* variants are known variants that have previously been reported (c.1132C>T [p.Q378X], c.1256G>A [p.R419Q], c.2542delG [p.V848CfsX83], c.3340C>T [p.R1114C], and c.4069C>T [p.R1357W]). c.1283A>G (p.N428S) is a variant of unknown significance as the p.N428S variant was only found in controls but not in patients with AS in a previous study [13]. However, in all three in silico programs, the p.N428S variant was predicted to cause severe damage to protein function. The remaining two variants, c.1939C>T (p.H647Y) and c.3374C>T (p.S1125F), have not been previously reported. The SIFT and PROVEAN programs predicted p.H647Y to cause severe damage, whereas the Polyphen-2 program predicted the variant to be benign. Regarding the variant p.S1125F, the in silico programs predicted it to cause severe damage to protein function. Neither of the p.H647Y and p.S1125F variants was found in the Single Nucleotide Polymorphism Database, the Human Genetic Variation database, the Exome Aggregation Consortium database, the Leiden Open Variation Database 3.0, or the Human Gene Mutation Database. Interestingly, segregation analysis in family 7 (JU#0481) revealed that two variants, p.N428S and p.H647Y, were on the same allele (Figure 2()).

### 3.2. Genotypes of the Patients with Angioid Streaks

Among the 18 families, *ABCC6* variants were identified in compound heterozygous or homozygous states in 13 probands and in heterozygous states in four probands, while no variants were identified in one proband (Table 1). Among the 13 families with disease-causing *ABCC6* variants, results were consistent with autosomal recessive inheritance in 11 families, while two families showed a pseudodominant inheritance pattern.  Figure 2 shows the pedigrees of the three families (families 3, 7, and 10) with the pseudodominant inheritance pattern and/or novel *ABCC6* variants (p.H647Y and p.S1125F). Twenty patients with AS in the current study had overlapping *ABCC6* variants, and some of these variants were found at a high frequency, including p.V848CfsX83 in 14 of 40 alleles (35.0%), p.Q378X in 7 of 40 alleles (17.5%), p.R1357W in 6 of 40 alleles (15.0%), and p.R419Q in 4 of 40 alleles (10.0%).

## 4. Discussion

In the current study, we identified eight disease-causing variants (p.Q378X, p.R419Q, p.V848CfsX83, p.R1114C, p.R1357W, p.N428S, p.H647Y, and p.S1125F) in the *ABCC6* gene, two of which (p.H647Y and p.S1125F) were novel variants. Thirteen of 18 AS probands (72.2%) exhibited homozygous or compound heterozygous states for the *ABCC6* variants, supporting the idea that AS is an autosomal recessive disorder.

Only one large-scale *ABCC6* gene analysis study has been previously reported; it was performed in 54 Japanese patients with AS, and six pathogenic variants (p.R419Q, p.E422K, p.V848CfsX83, a deletion of exon 23, c.3774_3775insC, and p.E1427K) were found [13]. Among those 54 patients, 17 patients (32%) carried homozygous or compound heterozygous variants, 17 patients (32%) carried heterozygous variants, and 20 patients (36%) did not carry any of the variants [13]. Notably, only two variants (p.R419Q and p.V848CfsX83) overlapped with our case series study. Our results showed high frequencies for some variants, including 14/40 alleles (35.0%) for p.V848CfsX83, 7/40 alleles (17.5%) for p.Q378X, 6/40 alleles (15.0%) for p.R1357W, and 4/40 alleles (10.0%) for p.R419Q (Table 2); of note, high frequencies for p.Q378X and p.R1357W were not found in the previous study [13]. Recently, Iwanaga et al. [14] reported an *ABCC6* mutation analysis study in 76 Japanese patients with PXE revealing that 56 (80%) of 76 patients had eye complications that were likely attributed to AS, while our study showed that PXE was seen in 15 of 17 AS patients (88.2%) who underwent dermatological examination. The pathogenic variant spectrum revealed five frequent variants: p.V848CfsX83 (34/152 alleles, 22.4%), p.Q378X (30/152 alleles, 19.7%), the deletion of exons 2 and 4 (15/152 alleles, 9.9%), p.Q199X (11/152 alleles, 7.2%), and p.R419Q (9/152 alleles, 5.9%) [14]. Of note, three variants (p.V848CfsX83, p.Q378X, and p.R419Q) were also frequently seen in our study. The *ABCC6* variants were identified to be in homozygous or compound heterozygous states in 13 of 18 families (72.2%) (Table 1). Two families (families 3 and 10) of the 18 families exhibited a pseudodominant inheritance pattern, and all four patients (cases 3, 4, 11, and 12) carried the p.V848CfsX83 variant (homozygously or compound heterozygously) that was detected the most frequent (Figure 2()). Taken together, these findings indicate that the *ABCC6* variants are likely a major cause of not only PXE but also AS in the Japanese population.

Our study revealed four frequent *ABCC6* variants (p.Q378X, p.V848CfsX83, p.R1357W, and p.R419Q) occupying 77.5% (31/40 alleles) of the total alleles. In contrast, two common recurrent variants (p.R1141X and del23_29) [7, 15] accounted for up to 40% of all variants in the European population [16, 17], but they were not found in the Chinese and Japanese populations [10, 13, 14], including in our study. These findings suggest that the *ABCC6* variant spectrum differs between Asian and European populations.

Two novel *ABCC6* variants (p.H647Y and p.S1125F) were identified in the current study. The p.H647Y and p.N428S variants were located on the same allele (Tables 1 and 2) in family 7 (JU#0481). The pathogenicity of the p.N428S variant had not been determined in a previous study because the variant was identified only in the control eyes [12]. In the present study, the in silico programs predicted that both variants (p.N428S and p.H647Y) cause severe damage to protein function. We concluded that both the p.N428S and p.H647Y variants on the same allele were variants of significance. The other novel variant, p.S1125F, was predicted by the in silico programs to cause severe damage to protein function, and case 11 (I-2 in family 10) carried the p.S1125F and p.V848CfsX83 variants and exhibited AS with PXE (Table 1), indicating the pathogenicity of p.S1125F.

The current study had several limitations, including case selection bias (as a single-center cohort), the small number of cases (*n* = 20), and our genetic analysis method in which deletion variants (such as deletion of exon 23 [13]) cannot be detected. In this study, four patients (cases 2, 10, 16, and 20) carried heterozygous variants, and one patient (case 9) did not carry any of the variants (Table 2); however, these five patients may have had a deletion variant, for example, the deletion of exon 23 [13] or the deletion of exons 2 and 4 [14], which cannot be detected using our Sanger-based methodology. A large cohort study with comprehensive clinical and genetic examinations will be necessary to clarify the genotype-phenotype correlations in patients with AS.

In conclusion, our results suggest that *ABCC6* variants play a significant role in patients with AS in the Japanese population and that four variants (p.Q378X, p.V848CfsX83, p.R1357W, and p.R419Q) are highly correlated with AS.

## Supplementary Material

Supplemental Table 1. The primer pairs and PCR conditions used for the screening of the *ABCC6* gene.

## Figures and Tables

**Table 1:** Clinical profiles and *ABCC6* variants of the patients with angioid streaks.

Family number	Case number	Gender	Age at examination	AS	CNV	PXE	Consanguinity	Genotype	Exon	Nucleotide changes	Amino acid changes	Notes
1 (JU#0376)	1	F	70	+	+	+	−	Homo	19	c.2542delG	p.V848CfsX83	
									19	c.2542delG	p.V848CfsX83	

2 (JU#0390)	2	F	49	+	−	+	−	Hetero	9	c.1132C>T	p.Q378X	

3 (JU#0446)	3	M	46	+	+	+	−	Compound hetero	9	c.1132C>T	p.Q378X	Father of case 4
									19	c.2542delG	p.V848CfsX83	
	4	F	16	+	−	+	−	Compound hetero	9	c.1132C>T	p.Q378X	Daughter of case 3
									10	c.1256G>A	p.R419Q	

4 (JU#0448)	5	M	57	+	−	+	−	Compound hetero	19	c.2542delG	p.V848CfsX83	
									29	c.4069C>T	p.R1357W	

5 (JU#0451)	6	F	45	+	+	+	−	Compound hetero	9	c.1132C>T	p.Q378X	Described in Figure 1
									29	c.4069C>T	p.R1357W	

6 (JU#0458)	7	F	60	+	+	+	−	Homo	9	c.1132C>T	p.Q378X	
									9	c.1132C>T	p.Q378X	

7 (JU#0481)	8	M	44	+	−	−	−	Compound hetero	10, 15	c.1283A>G, c.1939C>T	p.N428S, p.H647Y	
									19	c.2542delG	p.V848CfsX83	

8 (JU#0483)	9	M	51	+	−	ND	−	−	−	−	−	

9 (JU#0489)	10	F	23	+	−	+	−	Hetero	24	c.3340C>T	p.R1114C	

10 (JU#0554)	11	M	71	+	+	+	−	Compound hetero	19	c.2542delG	p.V848CfsX83	Father of case 12
									24	c.3374C>T	p.S1125F	
	12	M	36	+	−	+	−	Homo	19	c.2542delG	p.V848CfsX83	Son of case 11
									19	c.2542delG	p.V848CfsX83	

11 (JU#0602)	13	M	73	+	+	+	−	Compound hetero	9	c.1132C>T	p.Q378X	
									19	c.2542delG	p.V848CfsX83	

12 (JU#0476)	14	F	49	+	−	−	−	Compound hetero	19	c.2542delG	p.V848CfsX83	
									29	c.4069C>T	p.R1357W	
13 (JU#MD196)	15	M	77	+	+	ND	−	Compound hetero	19	c.2542delG	p.V848CfsX83	
									29	c.4069C>T	p.R1357W	

14 (JU#0627)	16	F	81	+	−	ND	−	Hetero	19	c.2542delG	p.V848CfsX83	

15 (JU#0630)	17	M	28	+	−	+	−	Compound hetero	10	c.1256G>A	p.R419Q	
									29	c.4069C>T	p.R1357W	

16 (JU#0639)	18	M	57	+	−	+	−	Compound hetero	19	c.2542delG	p.V848CfsX83	
									29	c.4069C>T	p.R1357W	

17 (JU#0651)	19	F	51	+	+	+	−	Compound hetero	10	c.1256G>A	p.R419Q	
									19	c.2542delG	p.V848CfsX83	

18 (JU#0653)	20	M	77	+	ND	+	−	Hetero	10	c.1256G>A	p.R419Q	

F = female; M = male; AS = angioid streaks; CNV = choroidal neovascularization; PXE = pseudoxanthoma elasticum; ND = not determined; homo = homozygous; hetero = heterozygous.

**Table 2:** Variants in the *ABCC6* gene identified in the 20 patients with angioid streaks.

Exon	Nucleotide	Amino acid	Homozygous	Heterozygous	Total (alleles)	dbSNP ID	HGVD (allele frequency)	ExAC East Asian (allele frequency)	ExAC total (allele frequency)	Polyphen-2 results (HumVar)	SIFT results	PROVEAN results	Pathogenicity	References
9	c.1132C>T	p.Q378X	1	5	7/40	rs72650699	0.002% (2/427)	0.000% (0/8646)	0.000% (1/121188)				Pathogenic	Le Saux et al. [2001], Pulkkinen et al. [2001]

10	c.1256G>A	p.R419Q	0	4	4/40	rs772434460	0.001% (2/1077)	0.001% (10/8204)	0.000% (10/111520)	0.742 (possibly damaging)	0.00 (damaging)	−3.27 (deleterious)	Pathogenic	Iwanaga et al. [2017]
	c.1283A>G	p.N428S	0	1	1/40	rs201880691	0.009% (20/1123)	0.001% (12/8524)	0.000% (13/117386)	0.999 (probably damaging)	0.00 (damaging)	−4.69 (deleterious)	Pathogenic	Sato et al. [2009] (reported in the controls)

15	c.1939C>T	p.H647Y	0	1	1/40	Not reported	Not reported	Not reported	Not reported	0.127 (benign)	0.01 (damaging)	−3.32 (deleterious)	Pathogenic	N/A (novel variant)

19	c.2542delG	p.V848CfsX83	2	10	14/40	rs67867306	Inconclusive	0.003% (27/8654)	0.000% (27/121324)				Pathogenic	Sato et al. [2009]

24	c.3340C>T	p.R1114C	0	1	1/40	rs63749794	Not reported	0.000% (0/8608)	0.000% (12/120184)	0.998 (probably damaging)	0.00 (damaging)	−7.13 (deleterious)	Pathogenic	Gheduzzi et al. [2004]
	c.3374C>T	p.S1125F	0	1	1/40	Not reported	Not reported	Not reported	Not reported	0.992 (probably damaging)	0.00 (damaging)	−4.85 (deleterious)	Pathogenic	N/A (novel variant)

29	c.4069C>T	p.R1357W	0	6	6/40	rs63750428	0.006% (14/1099)	0.001% (5/7944)	0.000% (6/107190)	1.000 (probably damaging)	0.00 (damaging)	−6.56 (deleterious)	Pathogenic	Miksch et al. [2005]

N/A = not applicable; HGVD = Human Genetic Variation database (http://www.hgvd.genome.med.kyoto-u.ac.jp/index.html); ExAC = Exome Aggregation Consortium database (http://exac.broadinstitute.org); Polyphen-2 (http://genetics.bwh.harvard.edu/pph2/); SIFT (http://sift.jcvi.org); PROVEAN (http://provean.jcvi.org).
