# Supplementary material for: ABCC6 Gene Analysis in 20 Japanese Patients with Angioid Streaks Revealing Four Frequent and Two Novel Variants and Pseudodominant Inheritance
Source: J Ophthalmol. 2017 Aug 20;2017:1079687. doi: 10.1155/2017/1079687 (PMC5585540; doi:10.1155/2017/1079687)
Supplement: Supplementary file 1 — Supplemental Table 1. The primer pairs and PCR conditions used for the screening of the ABCC6 gene. [file 1079687.f1.docx]

Supplemental Table 1. The primer pairs and PCR conditions used for the screening of the *ABCC6* gene

| Amplification region |  | Primer sequence (5´ to 3´) | Annealing temperature (°C) |
| --- | --- | --- | --- |
| Exon 1 (196 bp) | ABCC6-1F | CCGAGCAGTCTGCCCAGAGACTT | 64 |
|  | ABCC6-1R | GGGGGTCTCTCCTCTCCCCAGTAT |  |
| Exon 2 (311 bp) | ABCC6-2F | TGGCCCCCTGGGCAGGTGAG | 64 |
|  | ABCC6-2R | GTCCCCTGCCTCCCCCGAACA |  |
| Exon 3 (280 bp) | ABCC6-3F | CCGCCTACCAGTTTGCTGTGAC | 64 |
|  | ABCC6-3R | GGAGCCTCTTCTCTTCCCCTTGT |  |
| Exon 4 (388 bp) | ABCC6-4F | CTGCTGCTTTGCCTGCCACAGT | 64 |
|  | ABCC6-4R | GTGCGGGAGTGGATTTTGTGTCTCT |  |
| Exon 5 (295 bp) | ABCC6-5F | GTCCCCAGAGTGGGCACTGAC | 64 |
|  | ABCC6-5R | CTTTTGGTCACCTGGGGGAGAC |  |
| Exon 6 (283 bp) | ABCC6-6F | GGGCAACAGAGCGAAAACCTGTCT | 64 |
|  | ABCC6-6R | TGGAGAAAGACTTGCTGGCCTTTGT |  |
| Exon 7 (259 bp) | ABCC6-7F | GCCAGGATCCTGCAGGGGTGAA | 66 |
|  | ABCC6-7R | CGCACCCGGCCAATGATGAG |  |
| Exon 8 (327 bp) | ABCC6-8F | CCGCTGGCGGCTGAGAGTAT | 66 |
|  | ABCC6-8R | GGCCCTGGAAGGATGCCACTA |  |
| Exon 9 (4165 bp) | ABCC6-LR3F | CGACTGATCCTCCACATCTGGACTGGTTTG | 68 |
|  | ABCC6-LR2R | CGTCCACGGACACCAGATTGACCACATCAC |  |
| Exon 10 (440 bp) | ABCC6-10F | TTGTCATCTCTGTGGATCCTC | 62 |
|  | ABCC6-10R | GCCTCTTGAATGCTAAGTCAG |  |
| Exon 11 (253 bp) | ABCC6-11F | TGCTCTGGTTCACGTGCCTCTG | 64 |
|  | ABCC6-11R | TCAGCTCTCCCCTCCCCATCTC |  |
| Exon 12 (387 bp) | ABCC6-12F | GGTGAGATGAATGGGATTTGCTGAAG | 64 |
|  | ABCC6-12R | GGGGGGCTCCACCTACCTCAC |  |
| Exon 13 (286 bp) | ABCC6-13F | GCTTGCCCAGGCTGCCCTATC | 64 |
|  | ABCC6-13R | GGTAGGGAAGCTGGAGCCAGGTGTA |  |
| Exon 14 (262 bp) | ABCC6-14F | GCCACACATCTTGAGACACCGACAC | 64 |
|  | ABCC6-14R | CCAGTACTGATGCTGGCTTGCCATTA |  |
| Exon 15 (219 bp) | ABCC6-15F | ATGGTGCCTGGGGGCCTCTC | 64 |
|  | ABCC6-15R | GCAGGAGCCCCATGCATCTTCT |  |
| Exon 16 (282 bp) | ABCC6-16F | TCAGCTCCGTCTGGGGCTCATC | 64 |
|  | ABCC6-16R | GTGGGAAGGCAGCGAGGAAGTG |  |
| Exon 17 (343 bp) | ABCC6-17F | CAGCTCCCACTGCTCCTCAAAAC | 64 |
|  | ABCC6-17R | TCCATCATACTGCCCATGATGAGTC |  |
| Exon 18 (312 bp) | ABCC6-18F | AGCCTGGGCACCCCAGTTTC | 62 |
|  | ABCC6-18R | AAACTTGGGTTAGGACTGGATGCTAAGT |  |
| Exon 19 (374 bp) | ABCC6-19F | CCACATGCTTTGGCTTCCCAAAGTGT | 68 |
|  | ABCC6-19R | AGGGTGTGGCCAGAGCACTCCATTC |  |
| Exon 20 (221 bp) | ABCC6-20F | AAGGCCACATAGTCAGTGGGTGTCA | 68 |
|  | ABCC6-20R | GCGGGTGGTCCCTTCAGCTACT |  |
| Exon 21 (305 bp) | ABCC6-21F | TGGCTGTCAGTGGGCCTGAG | 66 |
|  | ABCC6-21R | GGTGAGTATCACTGCCAAGTGCTACA |  |
| Exon 22 (431 bp) | ABCC6-22F | TCCCATCTGCCATGGGCATGTTTT | 64 |
|  | ABCC6-22R | TTTGCACACTGTTCCAGGGGGACAG |  |
| Exon 23 (533 bp) | ABCC6-23F | CACCATGGGGTAGCGGGAGAGAC | 68 |
|  | ABCC6-23R | GGGAATTCTAGGAACAGCCCCTAGATGTC |  |
| Exon 24 (322 bp) | ABCC6-24F | GGCTCTCTGTGCTTCTGGAAACTA | 64 |
|  | ABCC6-24R | GGATATGGATGAATTGCAAGGTCTT |  |
| Exon 25 (270 bp) | ABCC6-25F | TCCTTGTGCCCAGAGAAGCATCTC | 68 |
|  | ABCC6-25R | CCACTAGCAGGGGTCCGACAGTC |  |
| Exon 26 (234 bp) | ABCC6-26F | CTCAAGTGGCCTTGCCAAACC | 64 |
|  | ABCC6-26R | GCCTGTAGCAGATGTCAACAG |  |
| Exon 27 (297 bp) | ABCC6-27F | GAAGCTGATAGAGGTGGGCCATCTTG | 64 |
|  | ABCC6-27R | GGTTTAGGGCCTTGTCCCTGGAGTC |  |
| Exon 28 (371 bp) | ABCC6-28F | GAGGGATGGATAGACAGATCTCGGGTACA | 64 |
|  | ABCC6-28R | ATCCGCAGAGAGCCAGGGAACAG |  |
| Exon 29 (296 bp) | ABCC6-29F | GGTGGAGGGGGTGGGCAAAGA | 64 |
|  | ABCC6-29R | GGCATGGCCATCCCCTCCTCTC |  |
| Exon 30 (344 bp) | ABCC6-30F | CTGTTTCTGGGCACACCCACACATC | 64 |
|  | ABCC6-30R | CCAGGACTGCCTCCGCCTCCT |  |
| Exon 31 (270 bp) | ABCC6-31F | CGCAGACACACTGGGCTCTCACA | 64 |
|  | ABCC6-31R | GATGACCACGGGTCACTTCCATCTC |  |

For exon 9 sequencing, we used either the ABCC6-9bF: TTGGGCAAAGGCAACACCCTTAG or ABCC6-9bR: ACTGCTTTTCCTGGCTGGGAAGAC primer.
